# Human Odour Coding in the Yellow Fever Mosquito, *Aedes aegypti*

**DOI:** 10.1038/s41598-019-49753-2

**Published:** 2019-09-16

**Authors:** Zhou Chen, Feng Liu, Nannan Liu

**Affiliations:** Department of Entomology and Plant Pathology, Auburn University, Auburn, AL 36849-5413 USA

**Keywords:** Neuronal physiology, Neuroscience

## Abstract

Insects use their olfactory systems to obtain chemical information on mating partners, oviposition sites and food. The yellow fever mosquito *Aedes aegypti*, an important vector of human infectious diseases, shows strong preference for human blood meals. This study investigated the chemical basis of host detection by characterizing the neuronal responses of antennal olfactory sensilla of female *Ae. aegypti* to 103 compounds from human skin emanations. The effect of blood feeding on the responses of olfactory sensilla to these odorants was examined as well. Sensilla SBTII, GP, and three functional subtypes of SST (SST1, SST2, and SST3) responded to most of the compounds tested. Olfactory receptor neurons (ORNs) ‘A’ and ‘B’ in the trichoid sensilla, either activated or inhibited, were involved in the odour coding process. Compounds from different chemical classes elicited responses with different temporal structures and different response patterns across the olfactory sensilla. Except for their increased responses to several odorants, blood-fed mosquitoes generally evoked reduced responses to specific aldehydes, alcohols, aliphatics/aromatics, ketones, and amines through the SST1, SST2, SBTI, SBTII and GP sensilla. The odorants eliciting diminished responses in female mosquitoes after blood feeding may be important in *Ae. aegypti* host-seeking activity and thus can be candidates for mosquito attractants in the process of this disease vector management.

## Introduction

Insects rely to a large extent on olfactory cues to locate mating partners, oviposition sites or hosts for food^1–3^. In particular, the mosquito *Aedes aegypti* (Linnaeus), a vector of many important human diseases including yellow fever, dengue fever and Zika fever, has been found to show strong preference for human hosts over other warm-blooded animals for blood meals^3,4^. At present, insecticides such as pyrethroids are the first choice for strategies to control mosquitoes, but the development of resistance to these chemicals has largely compromised their efficacy for mosquito management^5^. Alternative approaches based on the use of attractants and other lure-baited traps are therefore now widely used to control mosquito vectors^6,7^. Many compounds, including amines, carboxylic acids, ketones, and sulfides, have been shown to be effective in attracting mosquitoes, either on their own or to enhance the attractiveness of other chemicals, based on the results of behavioural assays^8^. CO_2_ has also demonstrated a remarkable synergistic effect on the attractiveness of single-compound lures, skin emanations and/or heat to mosquitoes^4,9,10^.

Female mosquitoes after a blood meal have shown reduced attraction to human hosts^11–13^. It has been suggested that decreased neuronal responses to certain human odorants may account for this diminished host-seeking activity^14^. The major olfactory organs of insects are their antennae, in which olfactory sensory neurons (OSNs) are located^15–17^. Odorant receptors (ORs), ionotropic receptors (IRs), and in some cases gustatory receptors (GRs) together with their highly conserved co-receptors form heteromeric ligand-gated ion channels on the membrane of OSNs in the antennae, thus contributing to the selectivity of odorant ligands in insects^1,15,17–19^. A blood meal has been shown to cause the down-regulation of specific olfactory receptor genes in hematophagous insects^13,20–23^, which may explain the decreased neuronal responses to certain human odorants in blood-fed individuals.

In this study, we characterized the neuronal responses of antennal olfactory sensilla of female *Ae. aegypti* to 103 compounds isolated from human skin emanations through GC-MS analysis^24^. We also explored the roles of olfactory receptor neurons (ORNs) ‘A’ and ‘B’ of trichoid sensilla in odour coding for the peripheral sensory system of *Ae. aegypti* mosquitoes, as well as the temporal dynamics of the neuronal responses elicited by structurally related compounds and how the spatial relationships between these compounds is represented in odour space. Finally, we examined the effect of blood feeding on the sensitivity of olfactory sensilla to human odorants in order to identify those that may be important in *Ae. aegypti* host-seeking activity.

## Results

### Responses of antennal olfactory sensilla to human odorants in *Ae. aegypti*

**Figure 1:**
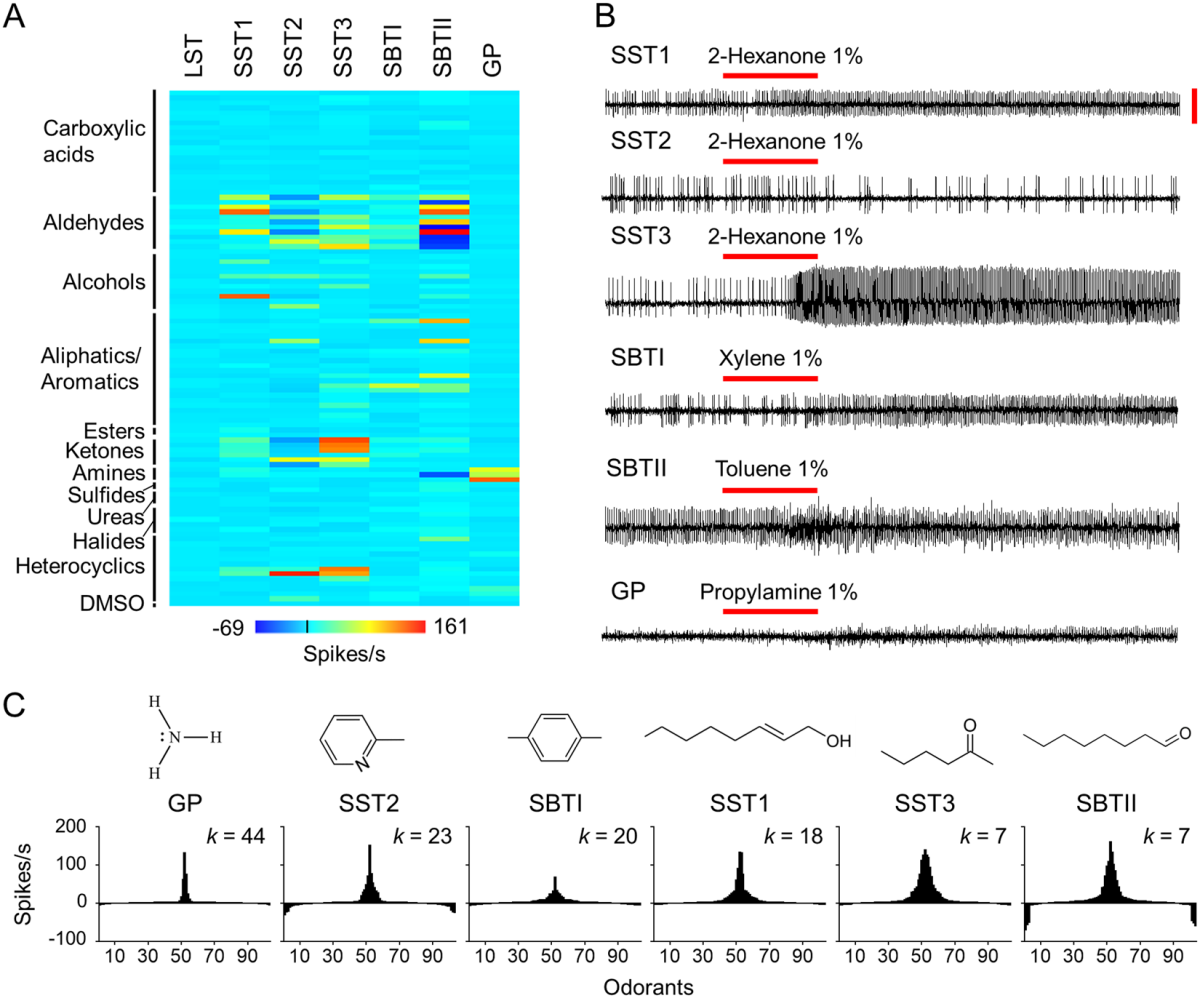
Functional characterization of antennal olfactory sensilla in *Ae. aegypti*. (**A**) Heat map of the neuronal responses of different types of sensilla to 103 human odorants. Compounds and numerical values are presented in Supplementary Table S2, n = 7–73. For compounds that elicited significant excitatory responses (≥15 spikes/s) or inhibitory responses (≤−10 spikes/s), *n* = 9–73. All odorants were tested at a dilution of 10^−2^, except that nonanal, heptanal, and octanal were examined against SBTII sensilla at dilutions of 10^−4^, 10^−3^, and 10^−3^, respectively, while ammonia was tested against GP sensilla at a dilution of 0.5 × 10^−3^. (**B**) Extracellular recording of sensillum neuronal response to a 500-ms pulse (horizontal red bar) of air through the odorant cartridge. Vertical red bar indicates 4 mV. (**C**) Tuning breadths of olfactory sensilla to human odorants. The 103 odorants are arranged along the *x*-axis according to the strength of the response they elicited in each type of sensilla. The odorants that elicited the strongest responses are placed near the centre of the distribution, while those that elicited the weakest responses are placed near the edges. The order of odorants is therefore different for each type of sensilla. The kurtosis value, *k*, indicates the ‘peakedness’ of each plot. Chemical structures of the odorants eliciting the strongest responses in each type of sensilla (ammonia (GP), 2-picoline (SST2), xylene (SBTI), trans-2-octen-1-ol (SST1), 2-hexanone (SST3), and octanal (SBTII)) are shown above each plot.

Five morphological types of olfactory sensilla have been previously identified on the antennae of *Ae. aegypti* mosquitoes, namely long sharp tipped (LST), short sharp tipped (SST), short blunt tipped I (SBTI), short blunt tipped II (SBTII), and grooved peg (GP)^25,26^. In this study, we examined the neuronal response of each type of olfactory sensilla of female *Ae. aegypti* mosquitoes against 103 human odorants from 11 chemical classes that have been isolated from skin emanations^24^. Hierarchical cluster analysis on the responses of these five morphological types of sensilla to the 103 human odorants revealed seven physiological clusters (Fig. S1). In particular, three functional subtypes of SST sensilla (55% of SST1, 14% of SST2, and 31% of SST3) were identified and defined according to their distinctive neuronal response profiles to the odorants on the panel (Fig. 1A,B). Sensilla SST1 and SST2 were reported in a previous study^27^.

In total, 721 odorant-sensillum combinations were tested for this study. Of these, 89 elicited significant excitatory (≥15 spikes/s) or inhibitory (≤−10 spikes/s) responses (Fig. 1A, Table S2). Specifically, 23 out of the 89 combinations yielded significant responses in the SBTII sensilla, followed by 19 in the SST3 sensilla, 17 in the SST2 sensilla, 16 in the SST1 sensilla, 9 in the SBTI sensilla, and 5 in the GP sensilla. The LST sensilla were not activated or inhibited by any of the human odorants included in the panel. All the aldehydes (11/11), ketones (6/6), and amines (3/3) elicited either excitatory or inhibitory responses in one or more types of sensilla (Fig. 1A, Table S2). The aldehyde-sensillum combination contributed to most of the odorant-sensillum combinations (37/89) that induced significant responses. However, only 7 out of 13 heterocyclics, 5 out of 13 alcohols, and 7 out of 23 aliphatics/aromatics activated one or more types of olfactory sensilla. None of the carboxylic acids, esters, sulfides, ureas, and halides tested elicited excitatory or inhibitory responses in any of the olfactory sensilla types. These findings suggest that female *Ae. aegypti* mosquitoes display bias toward detecting specific groups of human odorants such as aldehydes, ketones, and amines and these odorants may be involved in their host-seeking activities.

Different types of olfactory sensilla also exhibited differential preference in sensing human odorants. The tuning curves show that the GP sensilla were very selective, being narrowly tuned to five compounds (3 amines and 2 heterocyclics, with a *k* value of 44; Fig. 1A,C), and were thus defined as specialists. The SST3 and SBTII sensilla responded to more odorants, however, being tuned to 19 and 23 chemicals, respectively (7 aldehydes, 2 alcohols, 2 aliphatics/aromatics, 5 ketones, and 3 heterocyclics in the SST3 sensilla; 11 aldehydes, 2 alcohols, 6 aliphatics/aromatics, 2 ketones, 1 amine, and 1 heterocyclic in the SBTII sensilla; both with a *k* value of 7; Fig. 1A,C), and were thus regarded as generalists. Moreover, different types of sensilla gave their strongest responses to different odorants. For instance, the GP, SST2, and SBTI sensilla were tuned to ammonia (amine), 2-picoline (heterocyclic), and xylene (aliphatic/aromatic), respectively, while the SST1, SST3, and SBTII sensilla responded strongly to trans-2-octen-1-ol (alcohol), 2-hexanone (ketone), and octanal (aldehyde), respectively (Fig. 1C). In addition, although the SST1, SST3, and GP sensilla evoked only excitatory responses to the chemicals tested, the SST2 and SBTII sensilla evoked both excitatory and inhibitory responses (Fig. 1A,B).

### Integration of human odorant information via ORNs ‘A’ and ‘B’

**Figure 2:**
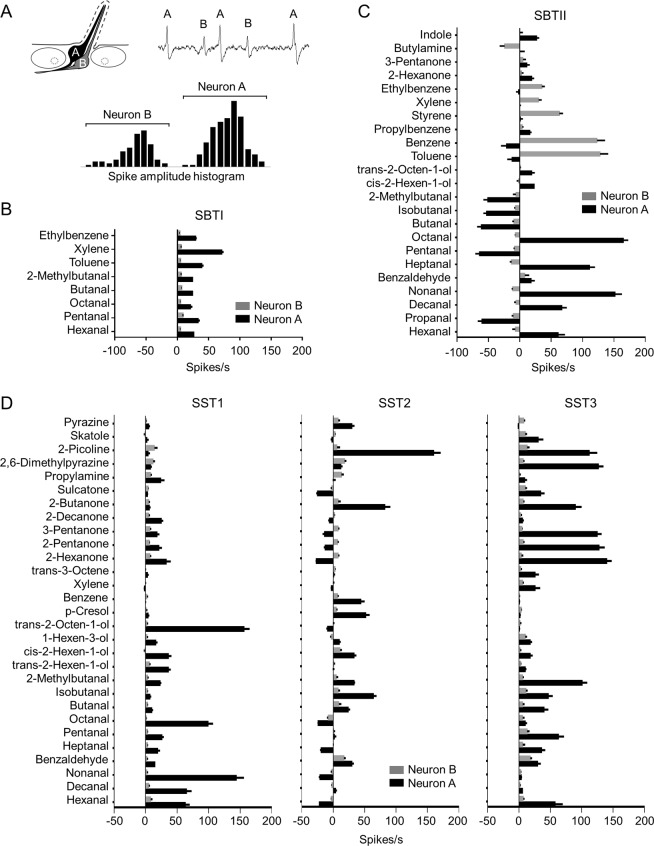
Comparison of response intensities for ORNs ‘A’ and ‘B’. (**A**) A trichoid sensillum that houses two ORNs, ‘A’ and ‘B’. Right: a single-sensillum recording of the spontaneous activities of both neurons. Action potentials from both ORNs can be distinguished by their amplitude, with the larger amplitude corresponding to Neuron ‘A’ and the smaller amplitude to Neuron ‘B’. Distribution of spike amplitudes of the two neurons is shown below. Response intensities for neuron ‘A’ (black bar) and neuron ‘B’ (grey bar) from sensilla SBTI (**B**), SBTII (**C**), and SST (**D**) are plotted. Only compounds that elicited significant responses (≥15 spikes/s or ≤−10 spikes/s) were used to analyse the response intensity for neurons ‘A’ or ‘B’. *n* = 7–13. Error bars indicate s.e.m.

Two ORNs were classified in the antennal trichoid sensilla of *Ae. aegypti* mosquitoes, which is consistent with the findings of a previous study^26^. Based on the histogram of spike amplitudes, the ORN with larger spike amplitudes was named as ‘A’ neuron, while the one with smaller spike amplitudes was assigned to ‘B’ neuron (Fig. 2A). The odorants that elicited significant excitatory (≥15 spikes/s) or inhibitory (≤−10 spikes/s) responses in each type of sensilla were chosen for the analysis of the response intensity of the ‘A’ and ‘B’ neurons. The results showed that the ‘A’ neuron alone was responsible for detecting all the aldehydes and aliphatics/aromatics that elicited excitatory responses in the SBTI sensilla (Fig. 2B), but both the ‘A’ and ‘B’ neurons in the SBTII sensilla were involved in sensing human odorants. The ‘A’ neuron in the SBTII sensilla was activated or inhibited by aldehydes, whereas the ‘B’ neuron was activated by aliphatics/aromatics (Fig. 2C). Interestingly, in the SBTII sensilla two aldehydes (nonanal and heptanal) activated the neuron ‘A’ but inhibited its neighbouring neuron ‘B’ (Fig. 2C). Although similar patterns were observed for toluene and benzene, in this case the outcomes were reversed, with the two aliphatic/aromatic compounds activating neuron ‘B’ but inhibiting its neighbouring neuron ‘A’ (Fig. 2C). Two alcohols (trans-2-octen-1-ol and cis-2-hexen-1-ol) and one heterocyclic compound (indole) also activated neuron ‘A’ in the SBTII sensilla, while the amine (butylamine) inhibited neuron ‘B’.

ORNs in all three functional types of SST sensilla showed distinctive patterns of activation or inhibition when exposed to the same panel of human odorants. Specifically, only neuron ‘A’ in the SST1 sensilla was triggered by aldehydes, alcohols, and ketones (Fig. 2D). In sensilla SST2, however, the situation was more complicated. For example, although neuron ‘A’ in the SST2 sensilla was inhibited by several aldehydes (hexanal, nonanal, heptanal, and octanal) and all ketones except for 2-butanone, it was activated by two alcohols (cis-2-hexen-1-ol and p-cresol), one aliphatic/aromatic (benzene), two heterocyclics (2-picoline and pyrazine), and several other aldehydes (benzaldehyde, butanal, isobutanal, and 2-methylbutanal) (Fig. 2D). In the same sensilla, neuron ‘B’ was fired by benzaldehyde and 2, 6-dimenthylpyrazine (Fig. 2D). Finally, compared to those in the SST1 and SST2 sensilla, neuron ‘A’ in the SST3 sensilla evoked more intense excitatory responses to ketones and heterocyclics (Fig. 2D). The neuron ‘B’ in the SST3 sensilla was activated by the aldehyde compound benzaldehyde (Fig. 2D). All these findings suggest that both ORNs ‘A’ and ‘B’ in the antennal trichoid sensilla of *Ae. aegypti*, whether activated or inhibited, are necessary for the detection of human odorants.

### Sensation of human odorants is dose-dependent

**Figure 3:**
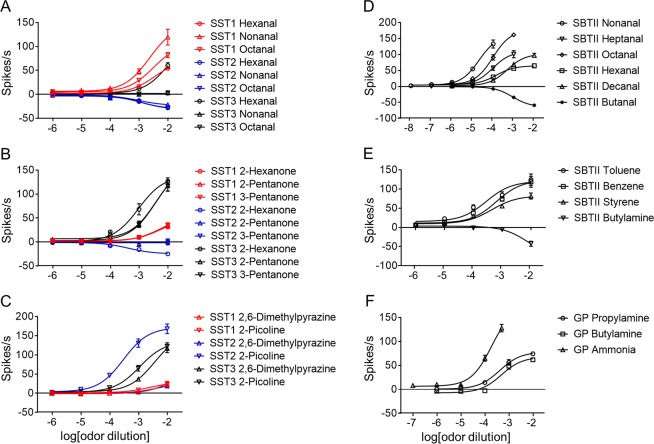
Dose-dependent responses of olfactory sensilla to human odorants. Aldehydes (**A**), ketones (**B**), and heterocyclics (**C**) tested in SST1 (red line), SST2 (blue line), and SST3 (black line) sensilla at serial 10^−2^–10^−6^ dilutions. (**D**) Aldehydes tested in SBTII sensilla. Nonanal tested at serial 10^−4^–10^−8^ dilutions; heptanal and octanal at serial 10^−3^–10^−7^ dilutions; hexanal, decanal, and butanal at serial 10^−2^–10^−6^ dilutions. (**E**) Three aliphatics/aromatics and one amine tested in SBTII sensilla at serial 10^−2^–10^−6^ dilutions. (**F**) Amines tested in GP sensilla. Propylamine and butylamine tested at serial 10^−2^–10^−6^ dilutions; ammonia tested at serial 0.5 × 10^−3^–10^−7^ dilutions. *n* = 5–7. Error bars indicate s.e.m.

To determine whether ORNs that responded strongly to certain odorants also responded with high sensitivity, we examined their responses to serial doses of compounds that elicited strong responses (≥50 spikes/s or ≤−10 spikes/s) at a 10^−2^ dilution. Three aldehyde compounds (hexanal, nonanal, and octanal) elicited dose-dependent excitatory responses in the SST1 sensilla but inhibitory responses in the SST2 sensilla (Fig. 3A). In the SST3 sensilla, ketones including 2-hexanone, 2-pentanone, and 3-pentanone induced stronger excitatory responses when their concentrations increased (Fig. 3B). 2-hexanone also produced dose-dependent responses in the SST2 sensilla but in an inhibitory manner (Fig. 3B). The SST2 sensilla showed a particularly high sensitivity to a heterocyclic compound (2-picoline), with a firing rate of 47 spikes/s at a 10^−4^ dilution (Fig. 3C).

Next, the sensitivities of the SBTII sensilla against aldehydes and aliphatics/aromatics and the GP sensilla against amines were tested. The SBTII sensilla were found to be very sensitive to nonanal, with a firing rate of 54 spikes/s at a 10^−5^ dilution, followed by octanal (24 spikes/s) and heptanal (16 spikes/s) (Fig. 3D). Butanal, however, produced dose-dependent inhibitory responses in the same sensilla. Three aliphatic/aromatic compounds (toluene, benzene, and styrene) induced excitatory responses in the SBTII sensilla, with the response increasing slowly with the concentration (Fig. 3E). One amine compound (butylamine) elicited inhibitory responses in the SBTII sensilla, but these responses did not follow a dose-dependent pattern (Fig. 3E). The GP sensilla showed high sensitivity to ammonia, with the response increasing sharply with the concentration (Fig. 3F). A further two amines also elicited excitatory responses in the GP sensilla, although the sensitivity of the GP sensilla to these two compounds was lower than their sensitivity to ammonia (Fig. 3F).

### Temporal dynamics of olfactory sensillum responses to human odorants

**Figure 4:**
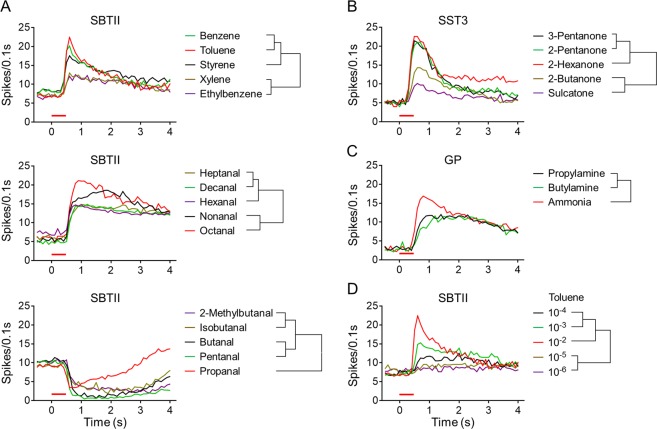
Temporal structures of neuronal responses to human odorants. Traces indicate the mean values of spikes (*n* = 7–9, error bars are not shown) recorded during each 100-ms sampling period, with the corresponding relationships within each group revealed by hierarchical cluster analysis. (**A**) SBTII sensilla responding to aliphatics/aromatics (upper) and aldehydes (middle and lower). All odorants were tested at a dilution of 10^−2^, except that nonanal, heptanal, and octanal were examined at dilutions of 10^−4^, 10^−3^, and 10^−3^, respectively. (**B**) SST3 sensilla responding to ketones at a dilution of 10^−2^. (**C**) GP sensilla in response to amines. All chemicals were tested at a dilution of 10^−2^, except for ammonia which was examined at a dilution of 0.5 × 10^−3^. (**D**) Toluene was examined in the SBTII sensilla at serial 10^−2^–10^−6^ dilutions. The horizontal red bar above the *x*-axis indicates a 500-ms pulse of stimulation.

Temporal dynamics may explain how an odorant is represented in the peripheral sensory systems of insects^28,29^. To investigate the temporal dynamics of the *Ae. aegypti* ORN responses to human odorants, strong responses induced by a 500-ms pulse of air through the compound cartridge were plotted to construct a set of temporal firing activities. Aliphatics/aromatics (including toluene, benzene and styrene) induced typical phasic responses in the SBTII sensilla, with the highest firing rates occurring 500 ms post-stimulus followed by rapid declines during the next 1.5-sec of the analysis period (Fig. 4A). When the same sensilla were challenged with certain aldehydes (octanal, nonanal, hexanal, decanal, and heptanal), typical tonic excitatory responses were observed and these responses were sustained throughout the 4-sec observation period (Fig. 4A). Other aldehydes elicited inhibitory responses in the same ORNs, but the temporal structures of these responses were different. In the SBTII sensilla, propanal induced significant phasic inhibitory responses, while other aldehydes (pentanal, butanal, isobutanal, and 2-methylbutanal) produced prolonged/tonic responses (Fig. 4A). Ketones also elicited more phasic responses in the SST3 sensilla (Fig. 4B), while three amine compounds (ammonia, propylamine, and butylamine) induced more tonic responses in the GP sensilla (Fig. 4C). Finally, the concentration of an odorant was found to influence the temporal structure of the response it elicited. For example, at a 10^−2^ dilution, toluene elicited a typical phasic response in the SBTII sensilla, but at lower concentrations it induced more tonic responses in the same sensilla (Fig. 4D). These results suggest that compounds from different chemical groups (and in some cases, those from the same group) may be represented differently in the peripheral sensory system of insects and this may be influenced by the concentration of an odorant that insects encounter.

### A primary presentation of odour space among the olfactory sensilla

To discover whether compounds from the same chemical group elicit similar ORN response patterns, the spatial relationships among the 39 human odorants yielding at least one response with a firing rate of ≥15 or ≤−10 spikes/s at a 10^−2^ dilution in any type of sensilla were examined in the odour space. The seven-dimensional (7D) odour space was constructed based on the primary responses of ORNs to the 39 compounds across seven types of sensilla. To quantify the relationships, the Euclidean distances between all possible pairs (a total of 741 pairs) of the 39 compounds were compared. Skatole and trans-3-octene (8 spikes/s), skatole and 1-hexen-3-ol (9 spikes/s), and 1-hexen-3-ol and trans-3-octene (9 spikes/s) were the three closest pairs, followed by 2-pentanone and 3-pentanone (12 spikes/s) and n-piperidineethanol and 4-peridinemethanamine (12 spikes/s). Of these, the compounds in the last two pairs were structurally related. Octanal and 2-picoline (264 spikes/s) and nonanal and 2-picoline (260 spikes/s) were the two pairs whose members were farthest apart in the odour space. The members of three additional pairs, octanal and isobutanal (256 spikes/s), octanal and pentanal (251 spikes/s), and nonanal and isobutanal (249 spikes/s) were also separated by large Euclidean distances, even though all the compounds involved came from the same chemical class.

**Figure 5:**
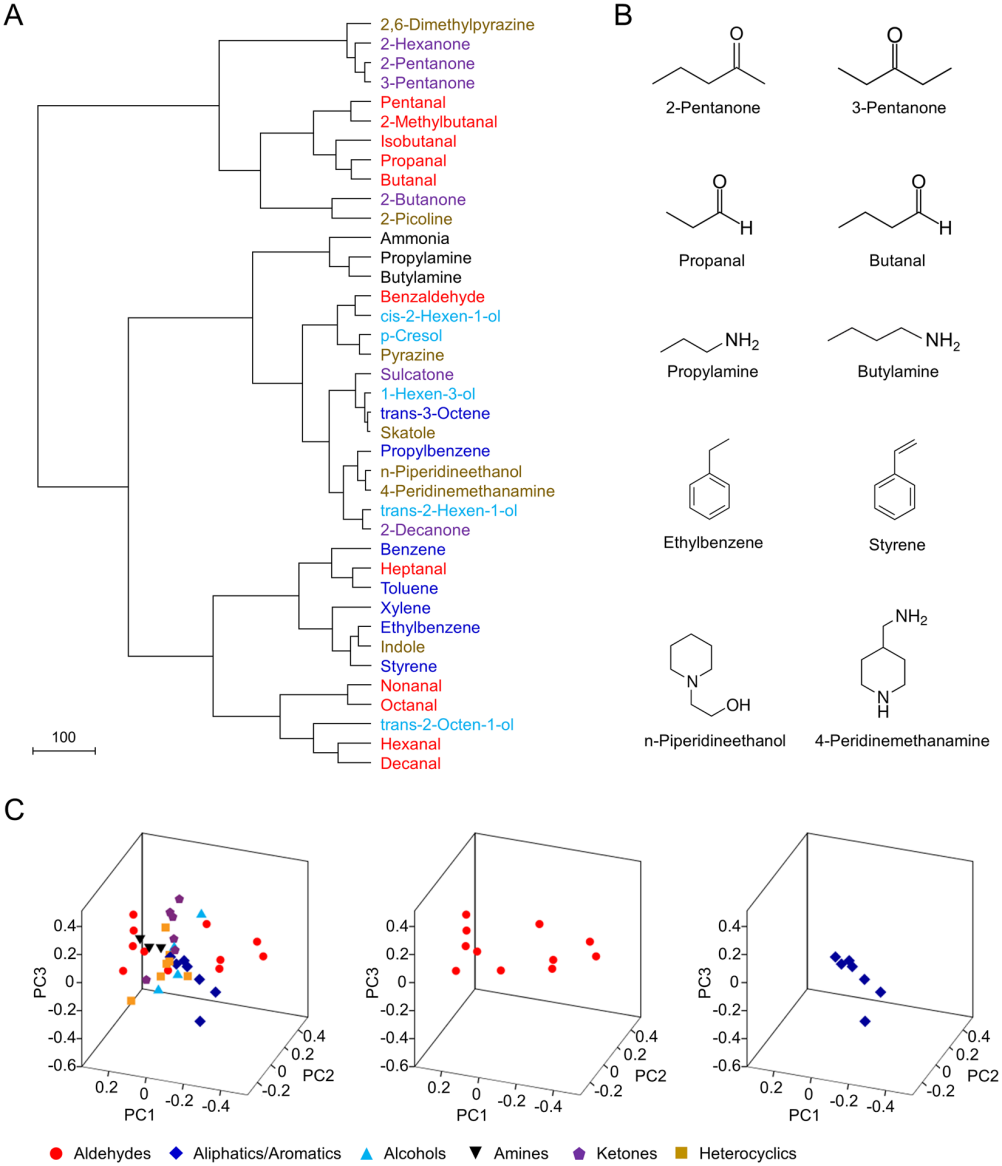
A primary presentation of odour space. (**A**) Hierarchical cluster analysis for 39 human odorants based on the Euclidean distances between odorants. These odorants elicited at least one response ≥15 spikes/s or ≤−10 spikes/s at a 10^−2^ dilution in any of the types of olfactory sensilla. Odorants are colour coded by chemical class. (**B**) Odorant pairs with close chemical structures are clustered together in the hierarchical cluster analysis. (**C**) Odour spaces as visualized by PCA for all 39 odorants (left), aldehydes alone (middle), and aliphatics/aromatics alone (right). By performing PCA for the neuronal responses of the seven types of olfactory sensilla to the 39 odorants, the original seven-dimensional odour space was represented in a three-dimensional space using the first three principal components. This three-dimensional representation accounts for 83.22% of the variance in the original seven-dimensional data set. Odorants are colour coded by chemical class, as shown below.

A hierarchical cluster analysis was then performed to visualize the spatial relationships among the 39 human odorants in the odour space. Though in no case was there a cluster which contained all members from the same chemical group, in general compounds with similar structures were tightly clustered together, such as 2-pentanone and 3-pentanone, propanal and butanal, and propylamine and butylamine (Fig. 5A,B). To represent the original 7D odour space in 3D space, principal component analysis (PCA) was carried out. In the 3D odour space, compounds from the aldehyde group were more widely dispersed from each other than those from the aliphatic/aromatic group (with a mean inter-odorant distance of 0.388 ± 0.023 for aldehydes and 0.205 ± 0.028 for aliphatics/aromatics; *P* < 0.001, two-tailed *t*-test), suggesting that compounds from the aldehyde group may elicit more variable response patterns across the seven different types of sensilla, while those from the aliphatic/aromatic group may induce more similar response patterns.

### Effect of a blood meal on the responses of olfactory sensilla to human odorants

Although 39 of the 103 human odorants tested elicited significant excitatory or inhibitory responses in the antennal olfactory sensilla of *Ae. aegypti*, this does not mean that all of them are necessarily involved in the host-seeking activity of *Ae. aegypti* mosquitoes. Female *Ae. aegypti* mosquitoes have shown decreased or no attraction to human arms at 24–72 hours post blood meal (pbm), but the strong attraction returns at 96 hours pbm^12^. This suggests that the attraction (or response) of blood-fed *Ae. aegypti* mosquitoes to certain human odorants may decrease at 24–72 hours pbm, and these odorants may be important in the host-seeking activity of *Ae. aegypti* mosquitoes.

**Figure 6:**
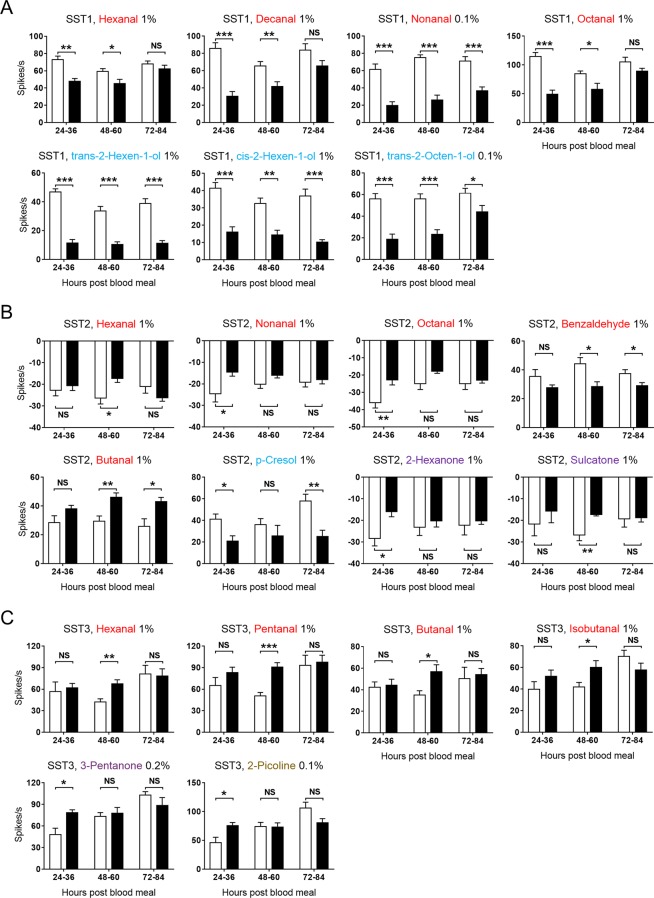
Effect of blood feeding on the neuronal responses of SST sensilla to human odorants. (**A**) SST1, *n* = 8–21; (**B**) SST2, *n* = 6–14; and (**C**) SST3, *n* = 6–10. Responses of olfactory sensilla in blood-fed (black bar) and non-blood-fed (white bar) female mosquitoes were compared at 24–36, 48–60, and 72–84 h pbm. Odorants are colour coded by chemical class, as shown in Fig. 5. An asterisk above the bar indicates: ***P < 0.001; **0.001 < *P* < 0.01; *0.01 < *P* < 0.05; NS, not significant, according to two-tailed *t*-test. Error bars indicate s.e.m.

To identify these salient human odorants, we compared the responses of antennal ORNs in blood-fed and non-blood-fed female *Ae. aegypti* mosquitoes to the 39 compounds. The results showed that the SST1 sensilla in blood-fed mosquitoes evoked significantly weaker responses to several aldehydes (hexanal, decanal, and octanal) at 24–36 and 48–60 hours pbm compared to those in non-blood-fed individuals (Fig. 6A). The SST1 sensilla of blood-fed mosquitoes also evoked reduced responses to another aldehyde compound (nonanal) and three alcohols, with the inhibitory effect of the blood meal lasting for up to 84 hours (Fig. 6A). The SST2 sensilla in blood-fed *Ae. aegypti* responded more weakly to certain aldehydes, alcohols, and ketones compared to those in non-blood-fed mosquitoes. For example, the SST2 sensilla of blood-fed mosquitoes evoked diminished inhibitory responses to three aldehydes (hexanal, nonanal, and octanal) and two ketones (2-hexanone and sulcatone), along with reduced excitatory response to benzaldehyde (Fig. 6B). However, the SST2 sensilla of blood-fed mosquitoes evoked a stronger response to butanal than those of non-blood-fed individuals at 48–60 and 72–84 hours pbm (Fig. 6B). A similar finding of enhanced ORN responses was also observed in the SST3 sensilla of blood-fed mosquitoes for four aldehydes (hexanal, pentanal, butanal, and isobutanal) at 48–60 hours pbm, one ketone (3-pentanone) and one heterocyclic (2-picoline) at 24–36 hours pbm (Fig. 6C).

**Figure 7:**
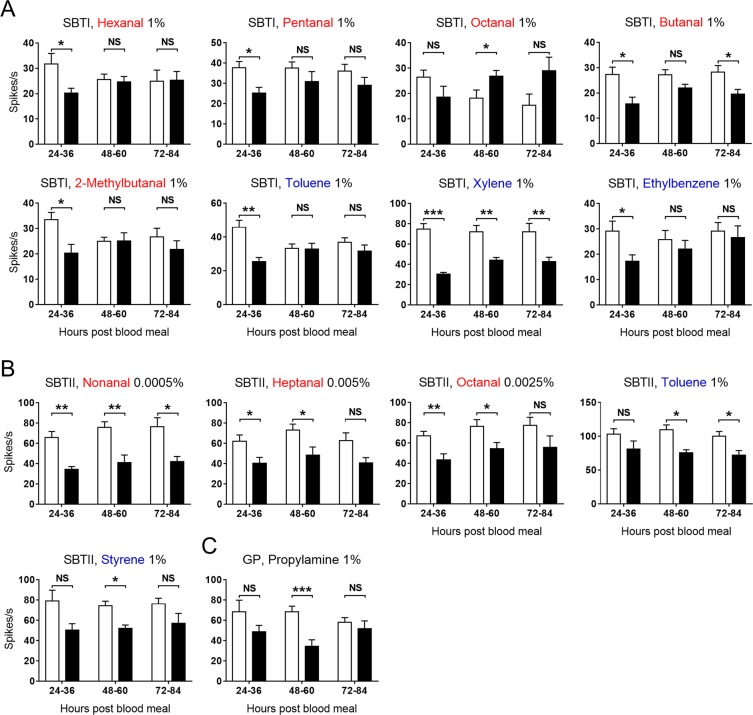
Effect of blood feeding on the neuronal responses of SBTI, SBTII, and GP sensilla to human odorants. (**A**) SBTI, *n* = 7–10; (**B**) SBTII, *n* = 7–22; and (**C**) GP, *n* = 11–20. Responses of olfactory sensilla in blood-fed (black bar) and non-blood-fed (white bar) female mosquitoes were compared at 24–36, 48–60, and 72–84 h pbm. Odorants are colour coded by chemical class, as shown in Fig. 5. An asterisk above the bar indicates: ***P < 0.001; **0.001 < *P* < 0.01; *0.01 < *P* < 0.05; NS, not significant, according to two-tailed *t*-test. Error bars indicate s.e.m.

The neuronal responses of the SBTI, SBTII, and GP sensilla to certain human odorants were also affected in mosquitoes after a blood meal. In comparison with non-blood-fed individuals, blood-fed mosquitoes evoked decreased responses to four aldehydes (hexanal, pentanal, butanal, and 2-methylbutanal) and three aliphatics/aromatics (toluene, xylene, and ethylbenzene) through their SBTI sensilla, though the response to octanal increased (Fig. 7A). The SBTII sensilla in blood-fed mosquitoes also showed compromised responses to specific aldehydes (nonanal, heptanal, and octanal) and two aliphatics/aromatics (toluene and styrene) at 24–36 or 48–60 (in some cases 72–84) hours pbm (Fig. 7B). However, the response of the GP sensilla in blood-fed mosquitoes to propylamine only reduced at 48–60 hours pbm (Fig. 7C).

## Discussion

In a previous study by our group, LST2, one of the two functional subtypes of LST sensilla in *Ae. aegypti*, was found to respond to two botanic terpenoid compounds (myrcene and terpinolene)^27^. However, the LST sensilla were not activated or inhibited by any of the human odorants on the panel used for this study. This may be because the LST sensilla of *Ae. aegypti* are narrowly tuned to specific compounds, including myrcene and terpinolene. The GP sensilla of *Ae. aegypti* responded to amine compounds, as reported previously on the same type of sensilla in other mosquito species^14,30^. IRs, another group of receptors responsible for odour sensation, and GRs, the group of receptors responsible for taste sensation, are thought to be involved in the detection of amines in insects^31–33^. As with ORs, IRs have been shown to be expressed in the antennae of *Anopheles gambiae* mosquitoes, though the exact sensillar localization of these IRs remains to be established^19^. However, GRs have been mapped in various locations in insects, including their antennae^15,17,34^. Carboxylic acids included in the panel did not induce responses in any types of antennal olfactory sensilla in *Ae. aegypti*, which is consistent with the findings reported in the southern house mosquito *Culex quinquefasciatus*^30^. Similarly, the *Drosophila* ORNs expressing *An. gambiae* ORs in the empty neuron system were not triggered by acids^29^, although IRs have been found to be responsible for acid sensation in the ORNs of coeloconic sensillum of *Drosophila*^35–37^. The reception of acids in mosquitoes needs to be examined in more detail.

How an odorant is encoded in the peripheral sensory system of insects remains largely unknown. In *Ae. aegypti*, we found that two aldehydes (nonanal and heptanal) activated the ‘A’ neuron of SBTII sensilla but inhibited its neighbouring ‘B’ neuron; whereas two aliphatic/aromatic compounds (toluene and benzene) activated the ‘B’ neuron of SBTII sensilla but inhibiting its neighbouring ‘A’ neuron. Interestingly, Su and her colleagues^38^ reported similar lateral neuronal inhibition in fruit flies and mosquitoes. In *Drosophila*, the ab3A neuron activated by methyl hexanoate was inhibited when its neighbouring ab3B neuron was activated by 2-heptanone or a pulse of light. In *Anopheles*, the cpA neuron activated by CO_2_ was also inhibited when its neighbouring cpB neuron was activated by 1-octen-3-ol. Unlike the lateral inhibition of ORNs found in *Ae. aegypti*, where it is caused by the same compound, the inhibition observed in both *Drosophila* and *Anopheles* results from a different stimulus. Moreover, we found that certain aldehydes and ketones (such as nonanal and 2-hexanone) activated the ‘A’ neuron of the SST1 and/or SST3 sensilla in *Ae. aegypti* while at the same time inhibited the ‘A’ neuron of the SST2 sensilla. These results suggest that coding of an odour in insects may require multiple ORNs from the same or different olfactory sensilla. Precisely how different ORNs process chemical signals at the peripheral sensory centre and how electrical signals are modified at higher olfactory processing centres awaits further investigation.

Previous studies have revealed the inhibitory effect of a blood meal on the host-seeking responses of female mosquitoes^11–13^. In our study, we found that blood-fed female *Ae. aegypti* showed decreased ORN responses to certain human odorants, including aldehydes, alcohols, aliphatics/aromatics, ketones, and amines on the panel. These compounds may thus be important in their host-seeking activities. Ammonia has been found to be effective in attracting *An. gambiae* females at low doses and has also been reported to enhance the attractiveness of lactic acid to both *Aedes* and *Anopheles* mosquitoes^39,40^. Specific ketones were also slightly attractive to female *Ae. aegypti*^41^. Interestingly, McBride and her colleagues^3^ found that the preference of *Ae. aegypti* for humans was associated with a high expression level of *Or4* and its high sensitivity to sulcatone (a ketone odorant from human skin emanation), although sulcatone perfumed with Guinea-pig odour was not preferred over the odour of Guinea-pig alone by human-preferring mosquitoes. Alcohols and aliphatics/aromatics were not attractive to *Ae. aegypti* on their own^41^. Aldehydes demonstrated little attractiveness or even repellence (at high concentrations) to female *Ae. aegypti* mosquitoes^41–43^. This suggests that a mixture composed of multiple human odorants (each at an optimized concentration) may be useful as an attractant for female mosquitoes.

The compromised responses to human odorants may be due to the down-regulation of certain olfactory receptor genes in blood-fed insects^13,20–23^. However, regardless of the overall decreased response levels, blood-fed female *Ae. aegypti* also evoked increased responses to four aldehydes, one ketone and one heterocyclic through their SST3 sensilla. The enhanced responses to these human odorants may result from the overexpression of specific olfactory receptor genes^13,21,23^, which may also respond to chemicals related to oviposition site cues. Blood-fed mosquitoes have been found to show increased ORN sensitivity to oviposition site cues, including heterocyclics (indoles), alcohols (phenols), ketones and carboxylic acids^14,44^. Taken together, a blood meal may exert a dual effect on the expression of olfactory receptor genes in insects, which finally modulates their ORN responses to cues from both hosts and oviposition sites.

## Materials and Methods

### Insects

*Ae*. *aegypti* mosquitoes (Orlando strain, obtained from Dr. James Becnel, USDA, ARS, Mosquito and Fly Research Unit) were maintained at 25 ± 2 °C and a photoperiod of 12: 12 (L:D) h (lights on 8 am). Females and males were reared together after eclosion and supplied with unlimited 10% sucrose solution throughout. For the experiment that characterized the neuronal responses of antennal olfactory sensilla to 103 human odorants, four- to six-day-old exclusively sucrose-fed female mosquitoes were used. For the experiment that examined the effect of a blood meal on the neuronal responses of antennal olfactory sensilla to human odorants, one group of female mosquitoes was fed with blood samples from horses (Large Animal Teaching Hospital, College of Veterinary Medicine, Auburn University) at the third day morning (8 am) after eclosion, while the other group was kept non-blood-fed and used as the control. Both blood-fed and non-blood-fed mosquitoes were thereafter supplied with unlimited 10% sucrose solution. The responses of the olfactory sensilla in both the blood-fed and non-blood-fed mosquitoes were tested against odorants at 24–36, 48–60, and 72–84 hours post blood meal (pbm).

### Electrophysiology

Extracellular single sensillum recording (SSR) was carried out as previously described^45^. Briefly, 4- to 6-day-old female mosquitoes were used after being anaesthetized on ice (1–2 min) and fixed with a 200 uL pipet tip. Mosquitoes were fixed by dental wax and a cover slip (22 × 22 mm) with double-sided tape. The reference tungsten electrode, connected to ground, was inserted into one eye of the tested mosquito and the recording electrode, which was connected to a preamplifier (Universal AC/DC Probe Gain 10×, Syntech), was inserted into the shaft of the test sensillum under a microscope (LEICA Z6 APO) using a micromanipulator (Leica, Cat #: 115378). The signal acquired by the preamplifier was digitized using an IDAC 4 (Syntech). Action potentials (i.e. spikes) evoked by a stimulus or control puff were recorded for 10 s, beginning 1 s before the stimulation. Action potentials were counted off-line for 500 ms before and after the stimulation. Firing rates recorded during the 500 ms post-stimulation period were subtracted from the spontaneous activities observed during the 500 ms pre-stimulation period and the outcome was multiplied by two to convert it to the conventional scale of spikes per second. Excitatory responses were recognized if the firing rate exceeded the response to the diluent control by 15 spikes/s and the inhibitory responses were identified as those where the firing rate diminished by 10 spikes/s or more^26^. The ORN evoking action potential with the larger amplitude was designated cell ‘A’, while the one yielding the action potential with the smaller amplitude was designated cell ‘B’^26^.

### Stimulation and stimuli

One hundred and three commercially available human odorants, identified through GC-MS analysis on skin emanations^24^, were used in the study (Table S1). These odorants fell into 11 chemical classes, namely carboxylic acids, aldehydes, alcohols, aliphatics/aromatics, esters, ketones, amines, sulfides, ureas, halides, and heterocyclics. Except for ammonia, which was diluted in ddH_2_O, the other odorants used as stimuli were freshly prepared every two weeks in dimethyl sulfoxide (DMSO) at the desired concentrations. For the dose-dependent assays, chemical stimuli were delivered to the sensillum under test from low to high dose and the delivery was performed as previously described^45^. In brief, 10 uL of each stimulant solution was dispersed onto a piece of filter paper (3 × 45 mm), which was then inserted into a glass Pasteur pipette and allowed to stand for 30 sec to create a stimulus cartridge. Ten microliters of 100% DMSO was used as the control stimulus. The preparation was bathed in a purified and humidified air stream (20 mL/s) flowing from a Stimulus Controller CS-55 (Syntech), to which the stimulus pulse at a rate of 0.5 L/min for 500 ms was added. For each odorant, each recording was from a separate sensillum. No more than three sensilla were tested per insect.

### Data analysis

Hierarchical cluster analysis using Euclidean distance and Ward’s method, heatmap construction, and principal component analysis (PCA) for odour space were performed with PAST 3.20 (University of Oslo). Tuning breadth curves and dose-dependent curves were fitted using GraphPad Prism 7.0. Statistical analysis was conducted by IBM SPSS Statistics (version 20, https://www.ibm.com/us-en/marketplace/spss-statistics). Error bars indicate s.e.m. unless otherwise noted.

### Use of experimental animals, and human participants

No live vertebrates and/or higher invertebrates were used in the experiments of the study.

## Supplementary information

SUPPLEMENTARY INFORMATION
