# Supplementary material for: Human Odour Coding in the Yellow Fever Mosquito, Aedes aegypti
Source: Sci Rep. 2019 Sep 16;9:13336. doi: 10.1038/s41598-019-49753-2 (PMC6746732; doi:10.1038/s41598-019-49753-2)
Supplement: Supplementary file 1 — SUPPLEMENTARY INFORMATION [file 41598_2019_49753_MOESM1_ESM.pdf]

Scientific Reports

SUPPLEMENTARY INFORMATION

Human Odour Coding in the Yellow Fever Mosquito, *Aedes aegypti*

Zhou Chen, Feng Liu & Nannan Liu*

Department of Entomology and Plant Pathology, Auburn University, Auburn, AL 36849-5413, USA

*To whom correspondence should be addressed: liunann@auburn.edu

1. **Table S1** Human odorants tested against the antennal olfactory sensilla in *Ae. aegypti*
2. **Table S2** Response profiles of different types of antennal olfactory sensilla to the human odorants tested (mean ± SEM, spikes/s)
3. **Figure S1. Hierarchical cluster analysis of the responses of five morphological types of olfactory sensilla.** Seven branch clusters suggest there are seven physiological types of sensilla, namely LST, SST1, SST2, SST3, SBTI, SBTII, and GP. Three to seven replicates were performed for each type of sensilla, as indicated by the numbers (1-6).

Table S1 Human odorants tested against the antennal olfactory sensilla in *Ae. aegypti*

**Chemicals***	**CAS number**	**Company**	**Purity (%)**	**Behavioural activity**	**SSR activity**	
					Antenna ORN	maxillary palp ORN
**Carboxylic acids**						
acetic acid	64-19-7	Sigma	99	[1-3]	[4-7]	
myristic acid	544-63-8	Sigma	98	[2, 8-9]		
hexanoic acid	142-62-1	Sigma	99.5	[2, 9]	[5-6, 10-11]	[12]
heptanoic acid	111-14-8	Sigma	96	[2-3]		[12]
propionic acid	79-09-4	Fisher	99.5	[1-3]	[4-6, 10]	
heptadecanoic acid	506-12-7	Sigma	98	[2]		
L-(+)-lactic acid	79-33-4	Sigma	98	[1-2, 9, 13-17]	[4, 10]	
n-pentadecanoic acid	1002-84-2	Sigma	99	[2]		
benzoic acid	65-85-0	Sigma	99.5	[2]		
trans-2,3-dimethylacrylic acid	80-59-1	Acros Organics	98			
DL-3-methylvaleric acid	105-43-1	Acros Organics	97			
octanoic acid	124-07-2	Sigma	98	[2, 9]	[5-6]	[12]
decanoic acid	334-48-5	Sigma	98	[2, 8]		
undecanoic acid	112-37-8	Sigma	98	[2]		
lauric acid	143-07-7	Sigma	99	[2, 8]		
n-tridecanoic acid	638-53-9	Sigma	98	[2]		
adipic acid	124-04-9	Acros Organics	99			
pimelic acid	111-16-0	Acros Organics	98			
4-hydroxybenzoic acid	99-96-7	Acros Organics	99			
acrylic acid	79-10-7	Acros Organics	99			
n-nonanoic acid	112-05-0	Sigma	97	[2-3, 8]		
**Aldehydes**						
hexanal	66-25-1	Sigma	98	[2, 9]		
propanal	123-38-6	Sigma	97			
decanal	112-31-2	Sigma	98	[2]	[18]	
nonanal	124-19-6	Aldrich	95	[2]	[18]	
benzaldehyde	100-52-7	Sigma	99			[12]
heptanal	111-71-7	Sigma	92	[2, 9]	[18]	
pentanal	110-62-3	Sigma	97			
octanal	124-13-0	Sigma	99	[2]	[18]	[12]
butanal	123-72-8	Sigma	99			
isobutanal	78-84-2	Sigma	99			
2-methylbutanal	96-17-3	Sigma	90			[12]
**Alcohols**						
2-decanol	1120-06-5	Sigma	98			
phenol	108-95-2	Sigma	99		[6]	
trans-2-hexen-1-ol	928-95-0	Acros Organics	96			
1-tetradecanol	112-72-1	Sigma	97			
2-hexadecanol	14852-31-4	Sigma	99			
cis-2-hexen-1-ol	928-94-9	Aldrich	95			
glycerol	56-81-5	Sigma	99	[2]		
1-hexen-3-ol	4798-44-1	Sigma	98	[9]		
1-octen-3-ol	3391-86-4	Aldrich	99	[3, 9, 13, 15, 17, 19]	[7]	[11, 16, 20]
trans-2-octen-1-ol	18409-17-1	Acros Organics	98			
4-ethyl phenol	123-07-9	Acros Organics	97		[6]	
p-cresol	106-44-5	Acros Organics	99			
phenelethyl alcohol	60-12-8	Sigma	99			
**Aliphatics/Aromatics**						

n-heptadecane	629-78-7	Sigma	99			
toluene	108-88-3	Sigma	99.8	[2]		
1-hexadecene	629-73-2	Aldrich	99			
1-tetradecene	1120-36-1	Aldrich	97			
n-nonane	111-84-2	Fisher	100			
benzene	71-43-2	Sigma	99.8	[2]		
squalene	111-02-4	Sigma	98	[2]		
propylbenzene	103-65-1	Sigma	98			
n-pentadecane	629-62-9	Acros Organics	99			
hexadecane	544-76-3	Acros Organics	99			
trans-2-octene	13389-42-9	Aldrich	97	[2]		
n-octadecane	593-45-3	Sigma	99			
styrene	100-42-5	Sigma	99	[2]		
n-decane	124-18-5	Fisher	99			
xylene	106-42-3	Sigma	99.5			
ethylbenzene	100-41-4	Sigma	99			
2,4-dimethylhexane	589-43-5	Fidher	99			
trans-4-octene	14850-23-8	Aldrich	98			
trans-3-octene	14919-01-8	Aldrich	98			
n-octane	111-65-9	Sigma	98			
2-pentene	109-68-2	Aldrich	99			
hexane	110-54-3	Sigma	95		[7]	[20]
heptane anhydrous	142-82-5	Sigma	99	[2]		
**Esters**						
methyl nonanoate	1731-84-6	Acros Organics	95			
methyl tridecanoate	1731-88-0	Acros Organics	97			
**Ketones**						
2-hexanone	591-78-6	Fluka	96			
2-pentanone	107-87-9	Fisher	99	[2]		
3-pentanone	96-22-0	Fisher	99	[2]		
2-decanone	693-54-9	Aldrich	98	[2]		
2-butanone	78-93-3	Sigma	99.7	[2]		[12]
sulcatone	110-93-0	Sigma	98	[2-3]	[21]	
**Amines**						
propylamine	107-10-8	Aldrich	99			
butylamine	109-73-9	Aldrich	99.5			
ammonia	1336-21-6	Aldrich	28	[2, 9, 22]		
**Sulfides**						
carbon disulfide	75-15-0	Fisher	99.9	[2]		
methyl disulfide	624-92-0	Sigma	99	[2]		
**Ureas**						
urea	57-13-6	Sigma	99			
methylurea	598-50-5	Sigma	97			
thiourea	62-56-6	Sigma	99			
**Halides**						
1-chloroheptane	629-06-1	Aldrich	99			
lauryl chloride	112-52-7	Acros Organics	99			
1-chlorotetradecane	2425-54-9	Acros Organics	98			
1-chlorohexadecane	4860-03-1	Aldrich	95			
1-chlorohexane	544-10-5	Fisher	95			
benzyl chloride	100-44-7	Sigma	99			
**Heterocyclics**						
indole	120-72-9	Aldrich	99	[2, 9]	[5-7, 11]	[12]
3-aminopyridine	462-08-8	Acros Organics	99			
4-aminopyridine	504-24-5	Acros Organics	98	[2]		
1-methylpiperazine	109-01-3	Acros Organics	99.5			

2-methylfuran	534-22-5	Acros Organics	99		
thiazolidine	504-78-9	Acros Organics	98		
2,6-dimethylpyrazine	108-50-9	Acros Organics	96		
2-picoline	109-06-8	Acros Organics	98		
skatole	83-34-1	Acros Organics	98	[9]	
coumarin	91-64-5	Acros Organics	99		
n-piperidineethanol	3040-44-6	Acros Organics	99		
4-peridinemethanamine	7144-05-0	Acros Organics	97		
pyrazine	290-37-9	Acros Organics	100		[12]
DMSO	67-68-5	Sigma	100		

*Human odorants were selected according to [23]. All odorants were tested at a dilution of 10^{-2}, except for nonanal, heptanal, and octanal in the SBTII sensilla, which were tested at dilutions of 10^{-4}, 10^{-3}, and 10^{-3}, respectively, while ammonia was tested in the GP sensilla at a dilution of 0.5×10^{-3}. Aldehydes (nonanal, heptanal and octanal) and amine (ammonia) at a dilution of 10^{-2} elicited such strong responses in the SBTII and GP sensilla that the responses were immediately inhibited upon delivery. Dimethyl sulfoxide (DMSO) was used to dilute all compounds except for ammonia, which was diluted in ddH2O. Numbers refer to previous behavioural studies or SSR studies on antennal/maxillary palp sensilla in *Ae. aegypti*.

Table S2 Response profiles of different types of antennal olfactory sensilla to the human odorants tested (mean ± s.e.m., spikes/s)

**Chemicals\ Sensilla**	**LST**	**SST1**	**SST2**	**SST3**	**SBTI**	**SBTII**	**GP**
**Carboxylic acids**							
acetic acid	4±2	2±1	2±1	-1±2	1±4	6±5	1±2
myristic acid	-2±2	0±1	0±1	8±3	3±3	6±2	3±2
hexanoic acid	1±2	3±2	1±2	5±4	1±2	6±2	2±2
heptanoic acid	1±1	-2±1	2±1	-2±3	-2±2	0±3	1±2
propionic acid	3±2	2±2	-2±2	2±5	2±4	1±4	1±2
heptadecanoic acid	2±2	3±1	0±2	4±3	0±2	-2±3	1±2
L-(+)-lactic acid	-2±2	3±1	1±2	4±3	0±3	12±6	0±2
n-pentadecanoic acid	2±1	1±1	-1±1	3±3	-1±2	10±3	1±2
benzoic acid	-1±2	0±2	1±2	2±2	-5±1	-1±3	-1±2
trans-2,3-dimethylacrylic acid	1±2	-1±2	2±1	2±4	2±2	0±2	3±2
DL-3-methylvaleric acid	-2±2	3±2	2±2	2±2	-2±2	0±3	2±1
octanoic acid	4±2	0±2	-2±2	-2±3	-2±2	-4±3	1±2
decanoic acid	3±2	2±2	0±2	4±2	3±3	2±3	1±1
undecanoic acid	-1±2	0±2	0±2	-2±3	2±3	1±2	0±2
lauric acid	-1±2	3±1	2±2	1±3	0±3	-2±3	0±2
n-tridecanoic acid	-2±2	0±1	-1±1	1±3	0±1	3±3	-2±2
adipic acid	0±2	1±2	0±2	0±2	4±3	-2±3	-1±1
pimelic acid	2±2	3±1	0±1	2±3	-3±1	1±4	3±2
4-hydroxybenzoic acid	1±2	0±1	-1±2	-2±2	-1±1	4±3	0±1
acrylic acid	1±2	1±1	3±2	0±3	7±3	-2±2	0±1
n-nonanoic acid	0±1	4±2	2±2	2±2	2±2	5±2	1±2
**Aldehydes**							
hexanal	0±3	60±3	-24±2	60±6	24±2	58±4	3±2
propanal	1±3	6±1	12±2	5±3	2±2	-49±4	2±2
decanal	7±3	75±4	-2±2	-2±3	13±9	85±6	-1±1
nonanal	1±2	131±6	-21±1	1±2	9±5	133±12	0±1
benzaldehyde	0±2	16±1	40±2	46±5	2±3	21±6	2±2
heptanal	3±2	13±2	-18±3	28±7	8±4	101±11	1±2
pentanal	7±3	16±2	2±2	69±7	31±3	-69±5	2±2
octanal	2±2	90±4	-29±2	12±4	18±2	161±6	2±2
butanal	-3±2	5±2	28±2	38±4	23±2	-59±5	1±2
isobutanal	3±3	4±1	71±4	42±5	1±2	-56±5	2±2
2-methylbutanal	0±2	13±3	32±3	93±8	24±2	-50±6	-1±2
**Alcohols**							
2-decanol	2±2	4±2	1±2	-3±1	-1±2	-4±2	3±2
phenol	2±2	0±1	2±4	3±3	0±2	4±3	2±2
trans-2-hexen-1-ol	1±2	32±2	-1±2	10±2	1±2	0±2	1±2
1-tetradecanol	1±2	1±1	1±1	0±2	3±1	7±3	1±2
2-hexadecanol	1±2	2±1	2±2	1±2	1±2	2±3	1±2
cis-2-hexen-1-ol	0±1	29±2	34±3	15±5	2±3	29±3	0±1
glycerol	3±2	-2±1	0±2	-1±2	1±2	-3±2	2±2
1-hexen-3-ol	3±2	6±2	3±2	30±3	0±1	8±5	3±2
1-octen-3-ol	1±1	0±2	-1±4	1±4	0±3	1±2	1±1
trans-2-octen-1-ol	-1±2	133±6	-7±2	1±2	0±2	17±4	3±3
4-ethyl phenol	1±1	-2±2	3±2	3±1	1±1	-2±2	0±2
p-cresol	3±2	0±2	48±3	2±2	-4±1	4±2	2±3
phenelethyl alcohol	4±2	1±1	3±2	2±2	3±3	0±3	1±1
**Aliphatics/Aromatics**							

n-heptadecane	4±2	1±2	4±2	1±2	-3±3	3±2	1±2
toluene	0±2	-2±1	1±3	8±4	33±3	108±4	1±2
1-hexadecene	3±2	2±1	4±1	2±2	3±2	1±3	-1±2
1-tetradecene	2±2	3±1	2±2	1±1	-2±1	-1±2	1±2
n-nonane	2±2	1±1	2±1	1±2	-2±1	-1±3	1±2
benzene	-3±2	-1±2	53±3	0±3	-1±2	97±6	0±2
squalene	1±1	3±2	3±2	-3±3	-1±2	-2±1	2±2
propylbenzene	3±2	0±2	-4±2	4±2	8±3	15±4	0±2
n-pentadecane	3±2	-1±1	0±2	2±2	5±3	5±3	2±2
hexadecane	1±2	0±2	1±2	-3±2	1±3	3±2	1±1
trans-2-octene	2±2	7±3	-1±3	3±3	1±2	2±3	-1±2
n-octadecane	0±1	0±2	3±2	1±2	-3±1	2±2	-1±1
styrene	-1±1	-2±2	0±2	14±5	8±5	71±3	4±2
n-decane	3±2	2±2	-3±1	0±2	3±2	2±2	1±2
xylene	3±3	0±2	-5±1	28±9	68±4	42±3	2±2
ethylbenzene	0±3	0±2	-6±1	13±5	26±2	40±2	-1±2
2,4-dimethylhexane	-1±2	2±1	1±1	5±2	-3±2	-1±2	0±2
trans-4-octene	2±2	0±2	-2±2	5±4	-1±3	2±2	1±1
trans-3-octene	3±2	3±2	1±2	23±7	3±3	6±3	1±2
n-octane	-1±2	0±2	0±2	1±2	2±3	1±1	0±1
2-pentene	1±2	1±2	1±1	12±4	-3±2	3±3	1±2
hexane	1±2	0±1	2±1	5±4	-2±2	4±2	2±2
heptane anhydrous	2±2	4±2	2±1	2±2	2±3	0±2	-2±1
**Esters**							
methyl nonanoate	2±2	12±3	-5±3	0±4	-2±4	5±5	1±2
methyl tridecanoate	4±2	5±3	0±2	4±5	0±2	2±2	1±2
**Ketones**							
2-hexanone	2±4	33±5	-22±2	139±5	14±2	18±4	-3±1
2-pentanone	-1±2	20±3	-9±2	125±8	3±3	7±2	0±1
3-pentanone	3±4	22±3	-6±2	120±8	9±4	15±4	1±2
2-decanone	2±4	27±4	-4±2	3±3	16±13	3±2	2±1
2-butanone	-3±2	5±2	77±3	75±7	1±3	1±3	0±1
sulcatone	2±2	2±2	-22±2	38±4	5±2	3±1	0±2
**Amines**							
propylamine	0±2	15±4	0±3	2±2	-1±3	2±2	75±4
butylamine	-2±2	12±4	5±3	5±4	5±4	-44±3	62±4
ammonia	2±2	1±1	2±2	-2±2	-2±2	2±2	132±6
**Sulfides**							
carbon disulfide	3±2	-2±1	1±1	-2±3	2±2	13±4	2±2
methyl disulfide	-1±2	-2±2	7±3	0±2	1±3	14±3	-1±2
**Ureas**							
urea	2±2	0±1	-1±2	0±2	6±6	5±2	3±3
methylurea	0±1	2±2	-2±2	-2±3	1±3	3±2	2±2
thiourea	0±2	2±1	2±2	2±2	0±2	6±3	1±2
**Halides**							
1-chloroheptane	0±2	-1±2	-4±3	0±2	7±5	7±4	3±2
lauryl chloride	0±2	1±1	-1±2	1±2	1±2	10±3	3±2
1-chlorotetradecane	7±2	0±2	3±1	5±2	1±3	4±1	-2±2
1-chlorohexadecane	-1±1	0±1	4±2	-3±3	3±2	4±3	0±2
1-chlorohexane	1±2	7±4	-4±1	-2±2	8±3	12±4	0±2
benzyl chloride	0±2	1±1	0±2	4±1	3±2	12±3	2±2
**Heterocyclics**							
indole	-1±2	1±2	3±2	2±3	2±3	42±3	0±2

3-aminopyridine	3±3	2±2	-1±2	0±2	1±2	1±3	1±2
4-aminopyridine	2±2	-2±1	4±2	4±2	5±2	1±3	0±2
1-methylpiperazine	2±2	1±1	3±2	3±2	-3±1	2±3	10±3
2-methylfuran	2±2	2±1	2±2	1±2	2±2	0±2	1±2
thiazolidine	2±1	-1±2	4±2	1±3	0±2	6±2	0±2
2,6-dimethylpyrazine	1±2	25±6	19±2	125±5	-1±2	10±4	-3±2
2-picoline	4±5	31±6	151±9	112±7	-1±1	8±3	5±3
skatole	3±2	-1±3	-1±1	30±5	4±3	6±2	1±2
coumarin	-1±2	1±1	-1±2	4±2	5±3	5±2	0±2
n-piperidineethanol	0±1	1±2	-1±2	-1±3	1±3	7±2	23±4
4-peridinemethanamine	1±2	-2±2	3±2	1±2	8±7	3±1	16±5
pyrazine	0±2	0±2	27±3	2±2	8±5	-2±2	2±2
DMSO	0±1	0±1	0±1	-1±1	1±3	-3±2	-1±1

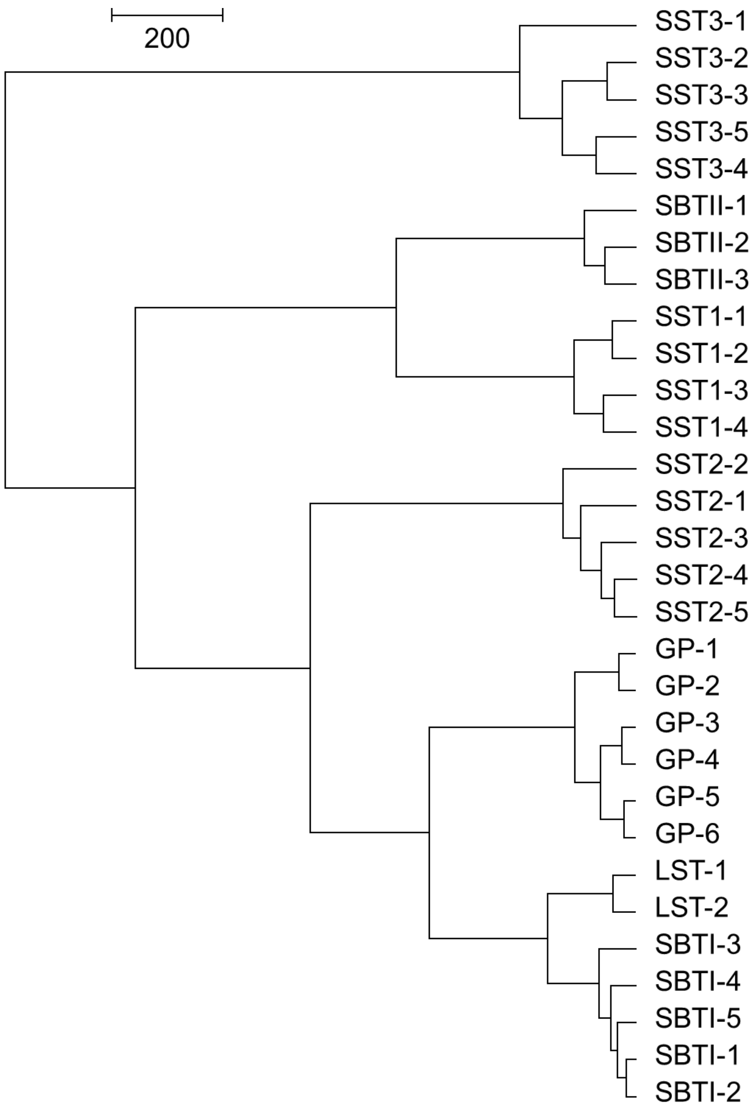

Figure S1. Hierarchical cluster analysis of the responses of five morphological types of olfactory sensilla. Seven branch clusters suggest there are seven physiological types of sensilla, namely LST, SST1, SST2, SST3, SBTI, SBTII, and GP. Three to seven replicates were performed for each type of sensilla, as indicated by the numbers (1-6).

References

1. Takken, W. The role of olfaction in host-seeking of mosquitoes: a review. *Int. J. Trop. Insect Sci.* **12,** 287–295 (1991).
2. Smallegange, R. C. & Takken, W. Host-seeking behaviour of mosquitoes: responses to olfactory stimuli in the laboratory. *Olfaction in vector-host interactions* (ed. Takken, W. & Knols, B.G.J.) 143-180 (Wageningen Academic, 2010).
3. Saratha, R. & Mathew, N. Development of a mosquito attractant blend of small molecules against host-seeking *Aedes aegypti*. *Parasitol. Res.* **115,** 1529–1536 (2016).
4. Davis, E. E. & Sokolove, P. G. Lactic acid-sensitive receptors on the antennae of the mosquito, *Aedes aegypti*. *J. Comp. Physiol. A Neuroethol. Sensory, Neural, Behav. Physiol.* **105,** 43–54 (1976).
5. Ghaninia, M., Ignell, R. & Hansson, B. S. Functional classification and central nervous projections of olfactory receptor neurons housed in antennal trichoid sensilla of female yellow fever mosquitoes, *Aedes aegypti*. *Eur. J. Neurosci.* **26,** 1611–1623 (2007).
6. Siju, K. P., Hill, S. R., Hansson, B. S. & Ignell, R. Influence of blood meal on the responsiveness of olfactory receptor neurons in antennal sensilla trichodea of the yellow fever mosquito, *Aedes aegypti*. *J. Insect Physiol.* **56,** 659–665 (2010).
7. Stanczyk, N. M., Brookfield, J. F. Y., Ignell, R., Logan, J. G. & Field, L. M. Behavioral insensitivity to DEET in *Aedes aegypti* is a genetically determined trait residing in changes in sensillum function. *Proc. Natl. Acad. Sci. U. S. A.* **107,** 8575–8580 (2010).
8. Ponnusamy, L. *et al*. Identification of bacteria and bacteria-associated chemical cues that mediate oviposition site preferences by *Aedes aegypti*. *Proc. Natl. Acad. Sci.* **105,** 9262–9267 (2008).
9. Mathew, N., Ayyanar, E., Shanmugavelu, S. & Muthuswamy, K. Mosquito attractant blends to trap host seeking *Aedes aegypti*. *Parasitol. Res.* **112,** 1305–1312 (2013).
10. Pappenberger, B., Geier, M. & Boeckh, J. Responses of antennal olfactory receptors in the yellow fever mosquito *Aedes aegypti* to human body odours. *Olfaction Mosquito-Host Interact* (ed. Bock, G. R. & Cardew, G.) 254–266 (Wiley, 1996).
11. DeGennaro, M. *et al*. *orco* mutant mosquitoes lose strong preference for humans and are not repelled by volatile DEET. *Nature* **498,** 487–491 (2013).
12. Tauxe, G. M., Macwilliam, D., Boyle, S. M., Guda, T. & Ray, A. Targeting a dual detector of skin and CO2to modify mosquito host seeking. *Cell* **155,** 1365–1379 (2013).
13. Canyon, D. V. & Hii, J. L. K. Efficacy of carbon dioxide, 1-octen-3-ol, and lactic acid in modified Fay-Prince traps as compared to man-landing catch of *Aedes aegypti*. *J. Am. Mosq. Control Assoc.* **13,** 66–70 (1997).
14. Williams, C. R. *et al*. Geographic variation in attraction to human odor compounds by *Aedes aegypti* mosquitoes (Diptera: Culicidae): A laboratory study. *J. Chem. Ecol.* **32,** 1625–1634 (2006).
15. Cook, J. I. *et al*. Enantiomeric selectivity in behavioural and electrophysiological responses of *Aedes aegypti* and *Culex quinquefasciatus* mosquitoes. *Bull. Entomol. Res.* **101,** 541–550 (2011).
16. McMeniman, C. J., Corfas, R. A., Matthews, B. J., Ritchie, S. A. & Vosshall, L. B. Multimodal integration of carbon dioxide and other sensory cues drives mosquito attraction to humans. *Cell* **156,** 1060–1071 (2014).

17. Vinauger, C., Lutz, E. K. & Riffell, J. A. Olfactory learning and memory in the disease vector mosquito *Aedes aegypti*. *J. Exp. Biol.* **217,** 2321–2330 (2014).
18. Ghaninia, M., Larsson, M., Hansson, B. S. & Ignell, R. Natural odor ligands for olfactory receptor neurons of the female mosquito *Aedes aegypti*: use of gas chromatography-linked single sensillum recordings. *J. Exp. Biol.* **211,** 3020–3027 (2008).
19. Majeed, S., Hill, S. R., Birgersson, G. & Ignell, R. Detection and perception of generic host volatiles by mosquitoes modulate host preference: context dependence of (R)-1-octen-3-ol. *R. Soc. Open Sci.* **3,** 160467 (2016).
20. Grant, A. J. & Dickens, J. C. Functional characterization of the octenol receptor neuron on the maxillary palps of the yellow fever mosquito, *Aedes aegypti*. *PLoS One* **6,** 2–7 (2011).
21. McBride, C. S. *et al*. Evolution of mosquito preference for humans linked to an odorant receptor. *Nature* **515,** 222–227 (2014).
22. Bosch, O. J., Geier, M. & Boeckh, J. Contribution of fatty acids to olfactory host finding of female *Aedes aegypti*. *Chem. Senses* **25,** 323–330 (2000).
23. Bernier, U. R., Kline, D. L., Schreck, C. E., Yost, R. A. & Barnard, D. R. Chemical analysis of human skin emanations: comparison of volatiles from humans that differ in attraction of *Aedes aegypti* (Diptera: Culicidae). *J. Am. Mosq. Control Assoc.* **18,** 186–95 (2002).
